# Categorical and coordinate processing in object recognition depends on different spatial frequencies

**DOI:** 10.1007/s10339-014-0635-z

**Published:** 2014-09-19

**Authors:** Ayako Saneyoshi, Chikashi Michimata

**Affiliations:** 1 Department of Psychology, Teikyo University, 359, Otsuka, Hachioji-shi, Tokyo, 192-0395 Japan; 2 Department of Psychology, Sophia University, 7-1, Kioicho, Chiyoda-ku, Tokyo, 102-8554 Japan

**Keywords:** Object recognition, Spatial frequency, Categorical and coordinate spatial processing

## Abstract

Previous studies have suggested that processing categorical spatial relations requires high spatial frequency (HSF) information, while coordinate spatial relations require low spatial frequency (LSF) information. The aim of the present study was to determine whether spatial frequency influences categorical and coordinate processing in object recognition. Participants performed two object-matching tasks for novel, non-nameable objects consisting of “geons” (c.f. Brain Cogn 71:181–186, 2009). For each original stimulus, categorical and coordinate transformations were applied to create comparison stimuli. These stimuli were high-pass/low-cut-filtered or low-pass/high-cut-filtered by a filter with a 2D Gaussian envelope. The categorical task consisted of the original and categorical-transformed objects. The coordinate task consisted of the original and coordinate-transformed objects. The non-filtered object image was presented on a CRT monitor, followed by a comparison object (non-filtered, high-pass-filtered, and low-pass-filtered stimuli). The results showed that the removal of HSF information from the object image produced longer reaction times (RTs) in the categorical task, while removal of LSF information produced longer RTs in the coordinate task. These results support spatial frequency processing theory, specifically Kosslyn’s hypothesis and the double filtering frequency model.

## Introduction

The visual system has been suggested to encode two kinds of spatial relations (Kosslyn 1987, 1994, 2006): categorical spatial relations, which refer to the discrete spatial relationships of visual primitives easily described by verbal locatives (e.g., “object A is above object B”); and coordinate spatial relations, which represent the precise spatial relationships of visual primitives relative to coordinate metric values (e.g., “object A and object B are 2 cm apart”).

Previous studies have proposed that categorical and coordinate spatial processing depend on high spatial frequency (HSF) and low spatial frequency (LSF) information processing, respectively (Kosslyn et al. 1992; Jacobs and Kosslyn 1994; Laeng et al. 2011; Michimata et al. 2011; Okubo et al. 2010). Jacobs and Kosslyn (1994) proposed a computational theory of spatial processing. In this theory, categorical relations would be more efficiently represented by information from neural units with small, non-overlapping, receptive fields that encode HSFs, while coordinate relations would be more efficiently represented by neural units with large, overlapping, receptive fields that encode LSFs. Their theory was based on the “coarse coding hypothesis” (Hinton et al. 1986; see Discussion). This computational theory has been supported by computer simulation (Kosslyn et al. 1992) and behavioral studies (Okubo and Michimata 2002, 2004). Okubo and Michimata (2002) showed that the advantage of the right hemisphere for coordinate spatial relationship processing disappears following LSF removal. Similarly, Okubo and Michimata (2004) indicated that the advantage of the left hemisphere for categorical spatial relation processing disappears following HSF removal. These results indicate that different SF ranges are processed by distinct neural substrates that correspond to different types of spatial information processing.

**Fig. 1:**
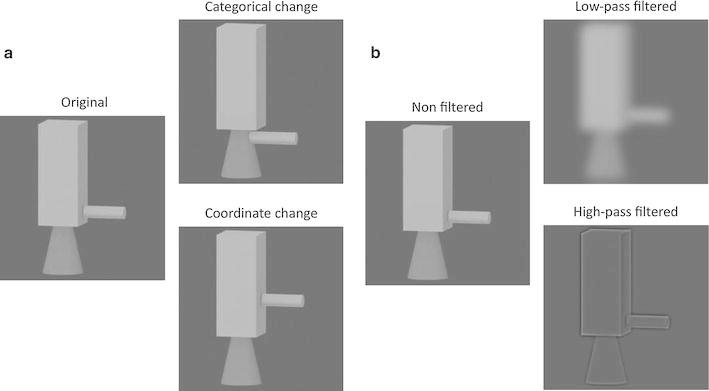
**a** Examples of the transformation pattern of stimuli used in the present experiment. *Left* original stimulus; *upper* an alternative with a categorical change in the arrangement of parts; *lower* an alternative with a coordinate change in the arrangement of parts. **b** Examples of the stimuli used in the present experiment. *Left* non-filtered, no SF manipulation; *upper* low-pass-filtered (high spatial frequencies were removed); *lower* high-pass-filtered (low spatial frequencies were removed)

Saneyoshi and Michimata (2009) concurred that these two forms of spatial processing may contribute to object recognition and proposed that the distinction between categorical and coordinate processing is helpful in understanding between-category and within-category object recognition, respectively. For example, between-category discrimination (e.g., cups vs. pots) requires consideration of the categorical spatial relationships of the parts (e.g., all coffee cups contain basic properties such as the attachment of a curved cylinder to the side of a main cylinder). Conversely, within-category membership discrimination (e.g., this cup vs. that cup) requires the processing of precise metric spatial relationships among the parts, as well as the use of metric figural features of the parts, such as length and curvature (e.g., different coffee cups may be distinguished based on the length of the main cylinder or the size of the curved cylinder; Biederman 1987; Cooper and Wojan 2000; Marr 1982). Saneyoshi and Michimata (2009) tested the role of categorical and coordinate processing using non-namable multipart objects consisting of three “geons” (c.f. Biederman 1987) as stimuli. In the categorical task, the original and categorical-transformed objects were used as the stimuli; in the coordinate task, the original and coordinate-transformed objects were used as stimuli. Categorical transformation consisted of transference of a geon from geon A to geon B (see Fig. 1a upper), whereas in coordinate transformation, a geon connected to geon A was moved to another position on geon A (see Fig. 1a lower). In both tasks, these objects were briefly presented one after another to the left or right visual fields, and participants judged whether they were the same or different. The results showed a left hemisphere advantage for categorical processing and a right hemisphere advantage for coordinate processing. Thus, Saneyoshi and Michimata (2009) successfully extended the categorical and coordinate processing hypothesis to multipart complex object recognition.

The purpose of the present study was to examine the hypothesis that the roles of the different SF ranges in spatial processing would extend to object recognition. In this study, we investigated whether low-pass/high-cut spatial filtering and high-pass/low-cut spatial filtering affect categorical and coordinate processing in object recognition. In this study, we employed the object set used in Saneyoshi and Michimata (2009) as stimuli. Further, these objects were spatially filtered using a high- or low-pass filter to remove low and high ranges of SF from images, respectively (see right panels of Fig. 1). We predicted that the removal of HSFs would reduce categorical processing, while the removal of LSFs would reduce coordinate processing in object recognition.

Although the stimuli used in this study and previous spatial relation studies bear some similarities, both consisting of compositions of simple elements, there are also marked differences. Our objects fundamentally differ from dot and bar stimuli in that the objects used in this study were volumetric and their views were changed by rotation. Furthermore, while the participants were required to discriminate the spatial relationships among elements (e.g., dot and bar) in the previous studies, the participants in this study were required to indicate whether the two objects were the same or different. Thus, the participants in this study were encouraged to recognize the stimuli as 3D volumetric objects. This study is also the first to investigate the role of spatial frequency in object recognition in terms of categorical and coordinate spatial relationships. Our predictions would be supported if there exist separate fundamental processing systems for categorical and coordinate visual information.

We also took the unconventional approach of informing participants about the nature of the stimulus manipulation, blocking the trials by task, and using different stimulus sets for different tasks. We wished to investigate top-down control of attention on the specific spatial frequencies that were hypothesized to be relevant for each spatial task so that it was crucial to block the trials by task and to inform the participants of the nature of the task.

## Methods

### Participants

Twenty-five undergraduate and graduate students (12 male and 13 female, age range: 18–24 years) volunteered to participate in this study. All had normal or corrected-to-normal visual acuity. They were unaware of the hypothesis under investigation. The participants gave their informed consent before participation.

### Apparatus

Stimuli were presented on a SONY 19-inch CRT monitor connected to an Apple Power Mac G4 computer that was running Mathworks Matlab 5.0 with PsychToolBox (Brainard 1997) software. A ten-key pad was connected to the computer and served as a response console.

### Stimuli

The tasks and stimulus features were consistent with Saneyoshi and Michimata (2009). First, the original grayscale pictures of 12 novel objects were created; each consisted of three component geons selected from four geons (cube, cylinder, cone, and sphere; Fig. 1a). Next, comparison stimuli were created by transforming each original picture. A categorical transformation consisted of the transference of a geon from geon A to geon B (Fig. 1a upper). A coordinate transformation consisted of the transference of a geon from one part to another part of geon A (Fig. 1a lower). For the categorical task, the stimulus set consisted of the original and categorically transformed objects, while for the coordinate task, the stimulus set consisted of the original and coordinate-transformed objects. All stimuli subtended approximately 4 × 4° of visual angle.

The distance of the displacement differed for each object and in each condition. For a given physical distance of displacement, the coordinate task was more difficult than the categorical task. Differences in task difficulty would affect the interaction between spatial frequency and task, so in order to equalize the task difficulties for categorical and coordinate change detection, the magnitude of displacements in the stimuli used in the two tasks were adjusted, using data from a pilot study. Thus, the magnitude of coordinate change was larger than the magnitude of categorical change for all objects. There was the possibility that the coordinate change would be considered as categorical change because of the large displacement of the parts. However, in the object classification test for the stimuli used in this study (see Saneyoshi and Michimata 2009), most of the coordinately transformed objects were judged as belonging to the same category of object. Thus, the coordinate-transformed objects would still be considered as coordinate changes, not categorical changes.

Following this, low-pass- and high-pass-filtered images (Fig. 1b) were created by computing Fourier transformations of the non-filtered image, convolving the output by using a filter with a 2D Gaussian envelope (*s* = 24 cpd for low-pass-filtered and *s* = 8 cpd for high-pass-filtered). These filtered Fourier images were subjected to inverse transformation. We used Matlab version 7.0 (Mathworks Inc., Natick, MA) for filtering manipulations. Further, for each object, two different views were created by rotating the object around the vertical axis approximately 10–20°; thus, the view of the second stimulus was always different from the view of the first in order to encourage recognition of the stimulus as a 3D volumetric object. In this manner, a total of 216 stimuli were created by an orthogonal combination of 12 objects, each with three transformations (original, categorical transformation, and coordinate transformation), three spatial frequency modifications (non-filtered, low-pass-filtered, and high-pass-filtered), and two views. Each image was presented twice (categorically or coordinate transformed) or four times (original, untransformed object) during one experiment so that it was difficult to predict or learn the change or the distance of displacement of stimuli.

## Experimental design

The experimental design was an orthogonal combination of Task (categorical vs. coordinate), and Spatial Frequency conditions [non-filtered, high-pass/low-cut filtering condition (hereafter referred to as high-pass condition) and low-pass/high-cut filtering condition (hereafter referred as low-pass condition)] and object match (Same vs. Different). All the variables were manipulated within participants. The dependent variables were error rates and reaction times (RTs).

On a given trial, two objects were presented sequentially. There were 72 pairs of the same object and 72 pairs of different objects. In both same-object and different-object pairs, the second stimulus of the pair was non-filtered in one-third of the trials (i.e., 48 trials; 24 of same pairs and 24 of different pairs). In a further third of the trials, the second stimulus was high-cut-filtered, and in the remaining third of the trials, the second stimulus was low-cut filtered. Thus, there were 144 trials for each task, which were divided into six blocks of 24 trials consisting of an orthogonal combination of 12 objects, three SF manipulations, two same–different conditions, and two views of the first stimulus for each task.

### Task and procedure

Participants were told to keep their right and left index fingers on the respective response keys. Participants were instructed to maintain their gaze toward the fixation cross and to respond as quickly and as accurately as possible. Participants were seated in a dark room approximately 114.8 cm away from the CRT monitor; their heads were positioned on a chin rest. In both tasks, two objects (original and transformed) were presented sequentially, and participants judged whether they were the same or different. The stimulus set for the categorical task consisted of the original and the categorical-transformed objects, while the set for the coordinate task consisted of the original and the coordinate-transformed objects. Participants were provided with a complete explanation about the nature of the transformation for each task and were instructed to attend to the appropriate aspect of component relations in order to perform the task. In each trial, a fixation cross appeared for 500 ms, followed by a 1,500 ms presentation of the first stimulus in the center of the visual display. The first stimulus was always non-filtered (i.e., included the full range of SF). After the offset of the first stimulus, a mask appeared for 500 ms; this was followed by the second stimulus, which was presented for 150 ms. The second stimulus was non-filtered, low-pass-filtered, or high-pass-filtered. Participants completed 36 practice trials prior to the experiment, and they received a short break after each block. Finger-response mapping, task order, and block order were counterbalanced across participants.

## Results

The data of two participants who could not perceive high-pass-filtered images and of one participant who showed a speed–accuracy trade-off (*r* = −.54) were deleted from the analysis. Thus, we analyzed data from 22 participants in total. For each participant, the percentage of errors and the median RT of correct responses were computed for each experimental condition. Error rates and RTs were subjected to a 3 (Spatial Frequency conditions) × 2 (Tasks) × 2 (Object match) repeated measures ANOVA. The correlation between RTs and error rates was positive (*r* = .262), suggesting there was no speed–accuracy trade-off. The alpha level was set to .05 for all statistics, and effect sizes are reported in terms of *η* ^2^ for ANOVAs.

**Fig. 2:**
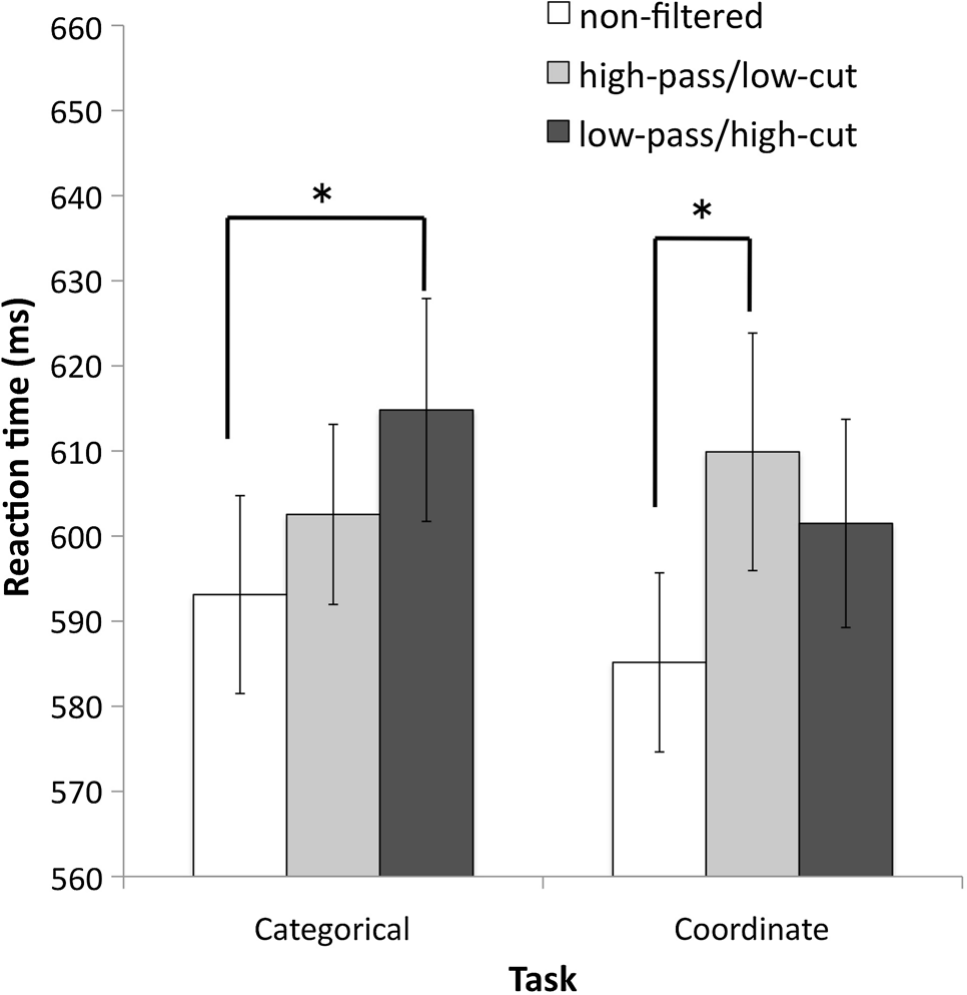
RT results for each experimental condition. *Gray bars* represent the intact condition; *white bars* represent the HSF condition; *black bars* represent the LSF condition. *Error bars* indicate 95 % confidence intervals calculated by the formula presented in Baguley (2012). *Asterisks* indicate significant pairwise comparisons

The RT results are presented in Fig. 2. There were no main effects of Task and Same–Different conditions (Task: *p* = .708, Same–Different: *p* = .630), or a Task × Same–Different × SF interaction (*p* = .060). The main effect of SF was significant [Sidak correction, *F*(1,21) = 8.44, MSE = 1141.96, *p* = .001, partial *η* ^2^ = .287], showing that the RT for the non-filtered condition (*M* = 589 ms, SE = 21 ms) was shorter than for both the high-pass (*M* = 606 ms, SE = 23 ms, *p* = .008) and low-pass (*M* = 608 ms, SE = 23 ms, *p* = .004) conditions. There was no difference between the high-pass and low-pass conditions (*p* = .976). Furthermore, the Task × SF interaction was significant [*F*(2,42) = 4.43, MSE = 572.15, *p* = .018, partial *η* ^2^ = .174]. Post hoc comparisons for this interaction revealed that in the categorical task, the low-pass condition (*M* = 614 ms, SE = 23 ms) produced longer RTs than the non-filtered condition (*M* = 593 ms, SE = 23 ms, *p* = .001). There were no differences between the high-pass (*M* = 602 ms, SE = 23 ms) and non-filtered conditions (*p* = .108) and between the high-pass- and low-pass-filtered conditions (*p* = .282). In the coordinate task, the high-pass condition (*M* = 610 ms, SE = 26 ms) produced longer RTs than the non-filtered condition (*M* = 585 ms, SE = 23 ms, *p* = .017). There were no differences between the low-pass condition (*M* = 601 ms, SE = 24 ms) and the non-filtered condition (*p* = .075) and between the low-pass condition and high-pass condition (*p* = .285).

Analysis of error rates revealed a significant main effect of Same–Different [*F*(1,21) = 5.96, MSE = 70.62, *p* = .024, partial *η* ^2^ = .221]. The error rate for the same condition (*M* = 3.60 %, SE = .62 %) was lower than for the different condition (*M* = 6.12 %, SE = .99 %). There were no main effects of Task or SF condition (Task: *p* = .716, SF: *p* = .212), and the Task × Same–Different × SF and Task × SF interactions were non-significant (*p* = .592 and *p* = .495, respectively). Because of the low overall error rate (mean = 4.86 %), there was no significant interaction in error rate.

Thus, the analysis of RTs revealed an interaction between Task and SF, which supports our prediction that HSF removal would deteriorate categorical task performance, while LSF removal would deteriorate coordinate task performance.

## Discussion

We predicted that the removal of HSFs would decrease categorical processing, while the removal of LSFs would decrease coordinate processing in object recognition. Our results showed a significant Task × SF interaction in the predicted direction. That is, multiple comparisons analysis revealed that a low-pass-filtered stimulus (i.e., removal of HSFs from the object image) produced a longer RT for the categorical task; however, there was no effect of a high-pass filter (i.e., removal of LSFs). This implies that HSFs are important for the processing of categorical properties in object recognition. In the coordinate task, a high-pass filter produced a longer RT, and there was no effect of a low-pass filter. This implies that the LSFs are important for the processing of coordinate (metric) properties in object recognition.

Our results are consistent with Kosslyn’s hypothesis (Kosslyn 1994) and previous spatial relation studies (Okubo and Michimata 2002, 2004), which show that different ranges of SF information are critical for the perception of differences in the visual world. While numerous studies on categorical and coordinate information processing have verified Kosslyn’s hypothesis by using simple dot and bar stimuli (Cowin and Hellige 1994; Okubo and Michimata 2002, 2004), we observed the same pattern in complex object recognition. Thus, Kosslyn’s hypothesis regarding spatial relations and the specific roles of different spatial frequency processing mechanisms can be extended to include object recognition.

Previous experiments have reported that the coordinate task is more difficult to perform than the categorical task. This difference in task difficulty may generate a concern that the interaction between task and other factors may be partially attributed to task difficulty (see Jager and Postma 2003). However, there was no main effect of Task in the non-filtered conditions in our study. The extent of the categorical and coordinate transformations was adjusted in our stimuli so that the two types of comparison object were equally difficult to process (see Methods in Saneyoshi and Michimata 2009). Thus, the absence of a Task main effect in the present results indicates that task difficulty did not account for the differential effect of categorical versus coordinate processing.

One might argue that the different discrepancy distances for the categorical and coordinate changes would impact the precision needed to perform each task and thus have a bearing on the results. In fact, the magnitude of coordinate change was larger than magnitude of categorical change. Thus, in physical terms, detection of the categorical changes required greater spatial precision than detection of the coordinate changes. However, our results suggested that the categorical processing required HSF, which would provide low spatial precision, whereas the coordinate processing required LSF, which would provide high spatial precision. Therefore, our results indicated that it is not the physical magnitude of displacement that is critical. Instead, the task demands for the type of object recognition decide the required spatial precision and the relevant range of spatial frequencies. This result strongly supports our initial hypothesis.

It may appear odd that coordinate (metric) information, which requires high-resolution representation, is represented by LSFs. However, the “coarse coding” hypothesis proposed by Hinton et al. (1986) may explain this discrepancy. In the coarse coding hypothesis, metric coordinate spatial relations are processed efficiently by large, overlapping receptive fields that effectively encode LSFs. According to this hypothesis, decoding through the population activity of several units with large, overlapping receptive fields yields more precise localization of object parts and object features than does the activity from each individual unit (Hinton et al. 1986). In contrast, categorical spatial relations are processed more efficiently by small non-overlapping receptive fields, which effectively encode HSFs, because these fields can divide space into discrete bins.

Further, recent studies suggest that scope of spatial attention may be modulated using information derived from neural units with large and small receptive fields (Borst and Kosslyn 2010; Laeng et al. 2011; Michimata et al. 2011; Okubo et al. 2010). In Michimata et al. (2011), participants made categorical or coordinate spatial judgments on the global or local elements of hierarchically organized shapes. This procedure was based on the finding that attending on a local level requires small-field spatial attention, while attending on a global level requires large-field spatial attention. When participants attended to the local elements of the shapes (i.e., attending to HSF information), the categorical task was performed better. On the other hand, when participants attended to the global elements (i.e., attending to LSF information), the coordinate task was performed better. Additionally, Borst and Kosslyn (2010) observed similar results to Michimata et al. (2011) by using the Navon figure as a stimulus. These attention studies indicate that the necessary range of spatial frequencies changes in accordance with task demands: top-down attentional modulations modify the receptive field size to encode the necessary range of spatial frequency information in object recognition to satisfy task requirements.

While our results are consistent with previous categorical and coordinate processing studies, they are inconsistent with several previous object recognition and spatial frequency studies. For example, Collin and McMullen (2005) insisted that HSFs are used for member-level object recognition, while LSFs are used for basic object recognition. In contrast, Saneyoshi and Michimata (2009) indicated that the categorical information of objects is used for object recognition of a basic-category level, while coordinate (metric) information would be used for object recognition at a member level. According to our results, HSFs are crucial for categorical information processing (basic-category level in Collin and McMullen 2005), while LSFs are crucial for coordinate (metric) information processing (member level in Collin and McMullen 2005) in object recognition. Thus, the results of Collin and McMullen (2005) are inconsistent with the present findings. This inconsistency might have been caused by differences in the definition of stimulus hierarchy. The stimulus set used in Collin and McMullen (2005) consisted of 36 subordinate-level, six basic-level, and two superordinate-level stimuli. For example, “car” was a basic-level category name, and “Volkswagen” was the subordinate-level name. Additionally, “insect” was a basic-level name and “ladybug” and “mosquito” were subordinate-level names. Although these classifications were appropriate, it is possible that the subordinate-level objects in their stimuli were discriminated by the categorical information processing that mediated the HSF information. For example, “Volkswagen” was defined by the company’s characteristic logo and consequently required HSF information processing. On the other hand, “ladybug” and “mosquito” were distinguished by their categorical figural differences (i.e., LSF information; the main part of the ladybug was a circle, while the main part of the mosquito was a narrow ellipse). Thus, consideration of the computational elements that satisfy the task demands are fundamental to understanding the relationship between SF and object recognition processing.

Furthermore, Vannucci et al. (2001) indicated that HSFs are crucial for the recognition of tools, while LSFs are crucial for the recognition of animals. However, it should be noted that tool discrimination is based on the difference in categorical properties, while animal discrimination is based on coordinate (metric) properties. Thus, the relationship between SF and semantic category would be redefined according to the difference in required non-semantic object properties (categorical or coordinate) in order to distinguish different semantic categories. Although previous studies insist that a different SF range is required to distinguish different semantic categories of object or engage in different levels of object recognition, there is a possibility that the role of different SF ranges in object recognition, particularly semantic, would result from differences in more primitive, non-semantic, figural information processing.

Kosslyn et al. (1992) reported that categorical and coordinate spatial relation judgments were performed more effectively in a neural network model with two separate subsystems than an unsplit network. They additionally found that coordinate judgment was performed better when the input was filtered through larger, overlapping receptive fields (i.e., LSF information), while categorical judgment was performed better when the input was filtered through smaller, less overlapped receptive fields (i.e., HSF information). Their network model indicated that the hemispheric asymmetries for categorical and coordinate spatial relations are based on the utilization of different SF ranges. In fact, human studies (Okubo and Michimata 2002, 2004) indicate hemispheric asymmetries in the roles of different SF ranges in categorical and coordinate spatial processing. Furthermore, our results support the double filtering frequency (DFF) theory (Ivry and Robertson 1998), which states that categorical and coordinate spatial relations are based on the processing of HSFs and LSFs, respectively. In DFF theory, visual attention selects a SF range from the incoming spectrum that is most fitting for the task demands at a first stage. This selected SF range is then sent to the right and left hemispheres; the left hemisphere processes the HSF information, while the right hemisphere processes the LSF information. The present study suggests that the usage of different SF ranges for categorical and coordinate spatial relation processing could be extended to object recognition. Thus, future studies should examine the hemispheric asymmetry of categorical and coordinate property processing in object recognition, and the differential roles of high and low SF ranges.
